# Influence of insulin and glargine on outgrowth and number of circulating endothelial progenitor cells in type 2 diabetes patients: a partially double-blind, randomized, three-arm unicenter study

**DOI:** 10.1186/s12933-014-0137-4

**Published:** 2014-10-11

**Authors:** Dimitrios Oikonomou, Stefan Kopf, Rüdiger von Bauer, Zdenka Djuric, Rita Cebola, Anja Sander, Stefan Englert, Spiros Vittas, Asa Hidmark, Michael Morcos, Grigorios Korosoglou, Peter P Nawroth, Per M Humpert

**Affiliations:** Department of Medicine I and Clinical Chemistry, University of Heidelberg, Im Neuenheimer Feld 410, 69120 Heidelberg, Germany; Roche Diagnostics Deutschland GmbH Mannheim, Mannheim, Germany; Department of Cardiology, University of Heidelberg, Heidelberg, Germany; Institute of Medical Biometry and Informatics, University of Heidelberg, Heidelberg, Germany; Stoffwechselzentrum Rhein Pfalz, Mannheim, Germany

**Keywords:** Insulin glargine, Type 2 diabetes, Endothelial progenitor cells

## Abstract

**Background:**

Endothelial progenitor cells (EPC) are bone marrow-derived cells which can undergo differentiation into endothelial cells and participate in endothelial repair and angiogenesis. Insulin facilitates this in vitro mediated by the IGF-1 receptor. Clinical trials showed that the number of circulating EPCs is influenced by glucose control and EPC are a predictor of cardiovascular death. To study direct effects of insulin treatment on EPCs in type 2 diabetes patients, add-on basal insulin treatment was compared to an escalation of oral medication aiming at similar glucose control between the groups.

**Methods:**

55 patients with type 2 diabetes (61.6±5.9 years) on oral diabetes medication were randomized in a 2:2:1 ratio in 3 groups. Patients were treated additionally with insulin glargine (n=20), NPH insulin (n=22) or escalated with oral medication (n=13). Number of circulating EPC, EPC-outgrowth, intima media thickness, skin microvascular function and HbA1c were documented at baseline and/or after 4 weeks and 4 months.

**Results:**

HbA1c at baseline was, 7.3+/−0.7% in the oral group, 7.3+/−0.9% and 7.5+/−0.7% in the glargine and NPH insulin respectively (p=0.713). HbA1c after 4 months decreased to 6.8+/−0.8%, 6.6+/−0.7% and 6.7+/−0.6%, in the oral, glargine and NPH insulin group respectively (p=0.61). FACS analysis showed no difference in number of circulating EPC between the groups after 4 weeks and 4 months. However, the outgrowth of EPCs as detected by colony forming assay was increased in the NPH insulin and glargine groups (29.2+/−6.4 and 29.4+/− 6.7 units respectively) compared to the group on oral medication (23.2+/−6.3, p=0.013) after 4 months of treatment. A significant decrease of IMT from 0.80mm (+/−0.14) at baseline to 0.76mm (+/−0.12) after 4 months could be observed in all patients only (p=0.03) with a trend towards a reduction of IMT after 4 months when all patients on insulin treatment were compared to the oral treatment group (p=0.06). Skin microvascular function revealed no differences between the groups (p=0.74).

**Conclusion:**

The study shows that a 4-month treatment with add-on insulin significantly increases the outgrowth of EPC in patients with type 2 diabetes mellitus.

**Trial registration:**

(Clinical Trials Identifier: NCT00523393).

## Introduction

Endothelial progenitor cells (EPCs) are immature bone marrow-derived cells which undergo differentiation into endothelial cells and participate in endothelial repair and neoangiogenesis [1,2]. Clinical trials have shown that the number of circulating EPC correlates with cardiovascular risk factors and is an independent predictor of cardiovascular death [3,4]. Patients with diabetes type 1 and 2 have a reduced differentiation and function of EPC in vitro [2,5,6].

Poor glycemic control and increase of glycated hemog-lobin level is associated with an increase in the risk of cardiovascular events [7], death [8,9] and for the development of retinopathy or renal failure [9]. Data from clinical trials imply that there might indeed be positive effects of insulin therapy for the microvasculature [10] while there seem to be limited effects on macrovascular events in the ORIGIN and UKPDS trials [11,12]. Insulin has previously been shown to have positive effects on micro- and macrovascular function in experimental clinical trials [13–16]. So far it is still not known, whether the improvement of glucose control by any available therapeutic strategy would have the same effects.

We were previously able to show that intensified insulin therapy in poorly controlled diabetes leads to increased numbers of CD34/CD133-positive progenitor cells that can also be considered as precursors of EPC [17]. In addition, we could show that insulin stimulates the in vitro outgrowth of EPC and tube formation of adult endothelial cells in an IGF-1 receptor dependent manner [18]. Hence, we hypothesized that insulin could improve the outgrowth and number of circulating EPC independent of glucose levels and thereby improve micro- or macrovascular function.

In this controlled trial it was therefore studied, whether early addition of the long acting insulin analogue glargine or NPH-insulin are capable of increasing the number and differentiation of EPC compared to an escalation of oral diabetes medication after 4 months of treatment. In addition, the effects of insulin on microvascular function as well as carotid intima media thickness were studied in this randomized and controlled setting.

### Research design and methods

73 patients with type 2 diabetes mellitus according to the American Diabetes Association (ADA) criteria were enrolled in this prospective partially double-blind, randomized, three-arm mono-center trial (EudraCT-Nr: 2006-006573-24). The study took place at the University Hospital in Heidelberg, Germany. The main inclusion criteria were treatment with oral diabetes medication, HbA1c values > 6.5% and ≤ 9% and age between 35 and 70 years. The primary endpoint of the trial was the relative change of number of circulating EPC 4 weeks after start of therapy compared to baseline as detected by FACS analysis. Secondary endpoints included: i) change of number of circulating EPC 4 months after start of therapy compared to baseline as detected by FACS analysis ii) change in circulating EPC as detected by in vitro outgrowth iii) intima media thickness iv) skin microvascular function (as measured by laser Doppler perfusion upon heat stimulation) v) long-term Glucose control (HbA1c) vi) short-term Glucose control (fasting glucose). Glitazone and erythropoietin treatment was not permitted during the trial since they were previously shown to influence EPC [19,20]. All other oral antidiabetics as well as lipid and blood pressure medications were allowed. Patients with statin treatment were allowed to enter the study, yet statins were not added during the 4 months of treatment due to known effect on EPC [21]. Patients were randomized in a 1:2:2 ratio in 3 groups. The first treatment arm, consisting of patients receiving oral medication only, was treated openly. The other two treatment arms, consisting of patients receiving bedtime Insulin once daily in addition to existing oral medication, were double blinded with glargine controlled by NPH insulin. Randomisation of patients and monitoring of the trial was performed according to the Standard Operating Procedures from the Coordination Centre for Clinical Trials (KKS) in Heidelberg. Ready to use NPH insulin or glargine pens were packed and blinded by the pharmacy of the University Hospital in Heidelberg according to the randomization list. Anamnestic data (Table 1) in addition to blood tests, and EPC quantification, were documented/performed at the entry visit (visit 1), after 4 weeks (visit 2) and after 4 months (visit 3). Whereas intima media thickness (IMT) and laser Doppler data were documented/performed at the entry visit (visit 1) and after 4 months (visit 3).

**Table 1:** **Baseline patient characteristics (visit 1)**

	**Oral**	**Glargine**	**NPH Insulin**	**all**	**p**
**N**	13	20	22	55	
**Age (yrs)**	64.1 +/−4.1	60.1 +/−7.3	61.5 +/−5.0	61.6 +/−5.9	0.168
**Gender (female/male;%)**	6 (46)/7 (54)	7 (35)/13 (65)	9 (41)/13 (59)	22 (40)/33 (60)	0.810
**BMI (kg/m** ^**2**^ **)**	30.1 +/−5.4	32.7 +/−6.0	31.8 +/−5.2	31.7 +/−5.5	0.417
**Diabetes duration (yrs)**	6.2 +/−4.0	8.7 +/−6.6	9.8 +/−7.2	8.5 +/−6.4	0.286
**Diabetic neuropathy (yes: n;%)**	3 (23)	7 (37)	7 (35)	17 (33)	0.690
**Diabetic nephropathy (yes: n;%)**	0	3 (16)	2 (10)	5 (9)	0.324
**Diabetic retinopathy (yes: n;%)**	1 (8)	2 (10)	2 (10)	5 (9)	0.974
**Systolic blood pressure (mmHg)**	142.5 +/−14.3	145.1 +/−14.8	141.2 +/−12.9	142.9 +/−13.8	0.666
**Diastolic blood pressure (mmHg)**	79.6 +/−11.6	80.0 +/−10.2	79.1 +/−8.1	79.5 +/−9.6	0.964
**HbA1c (%)**	7.3 +/−0.7	7.3 +/−0.9	7.5 +/−0.7	7.4 +/−0.8	0.713
**Fasting Glucose (mg/dl)**	150.7 +/−41.1	166.8 +/−50.1	165.9 +/−38.9	162.6 +/−43.5	0.534
**Serum Creatinin (mg/dl)**	0.8 +/−0.2	0.9 +/−0.2	0.8 +/−0.2	0.8 +/−0.2	0.451
**Triglycerides (mg/dl)**	206.4 +/−131.2	208.9 +/−168.7	215.2 +/−110.7	210.8 +/−136.5	0.981
**HDL-cholesterol (mg/dl)**	48.5 +/−18.2	44.2 +/−10.0	44.2 +/−12.8	45.2 +/−13.3	0.599
**LDL-cholesterol (mg/dl)**	97.9 +/−30.0	88.1 +/−39.6	99.0 +/−27.5	94.8 +/−32.6	0.559
**Hemoglobin (g/dl)**	14.2 +/−1.0	13.6 +/−1.4	14.1 +/−1.3	13.9 +/−1.3	0.363
**Leukocyte count /nl**	6.3 +/−1.3	7.1 +/−2.2	7.5 +/−1.8	7.1 +/−1.9	0.187
**Statins (yes: n;%)**	7 (54)	14 (70)	10 (45)	31 (56)	0.271
**Intima media thickness (mm)**	0.83 +/−0.13	0.79 +/−0.13	0.79 +/−0.17	0.80 +/−0.14	0.737
**Skin microvascular function (%)**	555.1 +/−223.5	408.4 +/−216.5	455.7 +/−171.8	462.0 +/−205.5	0.132

On the day of visit 1, all patients randomized into one of the groups receiving insulin were educated by a qualified diabetes nurse or a physician on the following topics: i) use of the insulin pen ii) injection techniques and dosage iii) hypoglycemia awareness and self treatment according to the guidelines of the German Diabetes Association [22]. Patients receiving oral medication were educated concerning hypoglycemia awareness and self treatment only. All patients entering this trial were supplied with a device for blood glucose self monitoring (Accu-Check Aviva®, Roche Diagnostics, Mannheim Germany). In patients randomized to receive either glargine or NPH insulin, therapy was initiated with subcutaneous injection of 4 international units at bedtime (usually around 10 p.m.). The dose was adjusted according to morning fasting glucose levels using a titration scheme that was handed out to each participant and aiming at fasting glucose levels < 100 mg/dl. Patients on oral medication were titrated to maximum dose using the following combinations: metformin alone, DPP4-inhibitors alone, sulfonylurea alone, metformin + DPP4-inhibitors, metformin + sulfonylurea, metformin + glinides, acarbose could be added to any of these combinations. All patients were provided with a diabetes diary at visit 1 in which daily insulin dose, daily morning fasting glucose self- measurements, one diurnal glucose profile weekly (4 pre-meal values) and possible hypoglycemic episodes were documented.

In order to discuss possible adverse events and to further adjust treatment telephone calls of the investigators with the participants took place 2 weeks and 2 months after the entry visit.

The study complied with the Declaration of Helsinki and was approved by the Ethics Committee of the University of Heidelberg (Ethic-Committee-No: AFm0-023/2007); all patients entered the study according to the guidelines after giving informed consent.

### Laboratory parameters

On the day of all three visit time-points, ~ 20 ml of venous whole blood was drawn from the participants for measurement of the following routine parameters: HbA1c, glucose, hemoglobin, leukocyte count, erythrocyte count, triglycerides, total cholesterol, HDL-cholesterol and LDL-cholesterol, electrolytes (Sodium, Potassium), liver transaminases (GOT, GPT), creatinine and urea. The samples for routine parameters were sent to the department of clinical chemistry for analysis within 60 minutes after blood was drawn.

### Quantification of EPC using FACS-analysis

On the day of all three visits, ~ 10 ml of venous whole blood was drawn from the participants and peripheral blood mononuclear cells (pBMCs) were isolated by density gradient centrifugation (lymphocyte separation; PAA Laboratories, Pasching, Austria). EPCs were characterized by flow cytometric analysis as previously described in detail [17] using anti-CD34-FITC (Miltenyi Biotec GmbH, Germany) and VEGFR-2-PE (R & D Systems, Minneapolis, MN, USA). All measurements were normalized for auto fluorescence and unspecific receptor binding using FITC- (Pharmingen BD Biosciences) and PE-labeled isotype controls. EPC were counted and analyzed using FACS Calibur cell sorter (Becton Dickinson, Heidelberg Germany) and Cell Quest Pro Software (Becton Dickinson, Heidelberg, Germany) and expressed as a percentage of gated mononuclear cells.

### In vitro/ex vivo outgrowth of EPC

The outgrowth assay of EPCs in vitro was performed by a standard method indicating clonogenic potential of circulating EPCs as described in detail [3,23,24]. In brief, mononuclear cells were isolated from 30–40 mL whole blood by density gradient centrifugation (lymphocyte separation; PAA Laboratories, Pasching, Austria). PBMCs were preplated in tissue culture flasks (Sarstedt, Nuembrecht, Germany) in Medium199 (Invitrogen, Karlsruhe, Germany) supplemented with 20% fetal calf serum (PAA Laboratories) and penicillin/streptomycin/glutamine (Bio Whittacker, Walkersville, MD, USA) to allow adhesion of fast-adherent, differentiated cells. After 48 h, nonadherent cells were counted, and 10^6^ cells were plated on 24-cell plates coated with 25 μg/mL fibronectin (Sigma, St. Louis, MO, USA) and cultured in the same growth medium (see above) without any additional growth factors. Medium was changed after 3 days. Outgrowth of EPCs was quantified by counting of colony-forming units (CFUs).

### Intima media thickness

Intima media thickness was detected as previously described [25,26] at visits 1 and 3 by ultrasound in the right and left common carotid artery approximately 1–2 cm distal of the carotid bulb. Measurements were taken at the far wall of the artery at 4 different loci and in the end-diastolic phase of the heart cycle as documented by real time electrocardiogram.

### Skin microvascular function using laser-doppler

Microvascular responsiveness was studied at visit 1 and 3 using laser Doppler perfusion measurement with PeriFlux System 5000 (Perimed, Järfälla, Sweden) upon heat provocation as described in detail [27]. For this, 2 probes were placed on the dorsum of the right foot. Basic capillary perfusion was documented at 37°C skin temperature for 10 minutes and at 44°C for additional 10 minutes to study heat-provoked increase in perfusion as a marker of microvascular function. The data were stored on a computer and analyzed offline with signal processing software (PeriSoft 2.50, Perimed). No current laser Doppler instrument can provide absolute perfusion values. Measurements are expressed as Perfusion Units (PU), which are arbitrary.

### Statistical Analysis

According to [17] and assuming a 0.01, 0.05, and 0.04 per cent of peripheral mononuclear cells change in number of circulating EPC after 4 weeks in the oral treatment group, glargine, and NPH-insulin group, respectively, with a common standard deviation of 0.04 randomisation of 75 patients was initially planned to achieve a power of 80% with a two-sided global F test with alpha of 5%. The trial was stopped after 73 patients, erroneously assuming that the full sample size of 75 patients had been obtained. With the patient number in the intention to treat (ITT) population (n = 55) a power of 69.9% is reached under the initial assumptions. Pair-wise group comparisons were planned to be carried out according to the closed-testing principle only after showing significance in the global test. The confirmatory analysis was primarily based on the ITT population. If a patient discontinued the trial prematurely, missing data were not replaced. In addition to the evaluation of the ITT population, a per-protocol (PP) analysis was performed including all randomized patients without major protocol violations as sensitivity analysis. Secondary variables were tabulated using appropriate descriptive measures of the empirical distributions and descriptive P values for treatment group comparisons.

For evaluation of the primary endpoint, a global F-test was performed to test for differences between the three groups. No pair-wise group comparisons could be carried out. Secondary endpoints were analysed using descriptive measures and graphical approaches. Homogeneity of treatment groups was described by comparison of demographic data and baseline values. Comparisons of binary data were carried out using Pearson’s chi square test. In case of three groups a global F-test was performed, two groups were compared using the t-test. All additional evaluations were described and fixed in the statistical analysis plan before database closure. All analyses were performed using SAS® 9.2 (SAS Inc., Cary/NC, USA).

## Results

**Figure 1:**
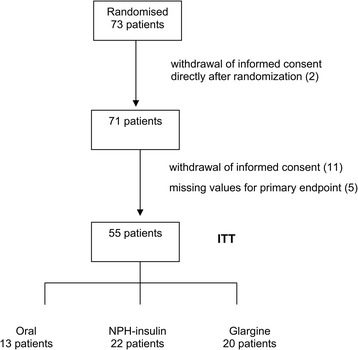
**Study flow-chart.**

10 out of 73 randomized patients stopped the trial before the first visit and therefore no values for EPC were obtained. 3 patients discontinued before visit 2, 4 patients had missing values for EPC after 4 weeks and one patient had a value of 0.00 and methodological bias at visit 1. Therefore the intention to treat population included 55 patients; 13 patients in the group with oral medication; 20 patients receiving insulin glargine and 22 patients receiving NPH Insulin (Figure 1).

HbA1c at baseline was 7.3+/−0.7%, 7.3+/−0.9% and 7.5+/−0.7% in the oral, glargine and NPH insulin group respectively and didn’t show any significant difference between the groups (p = 0.713; Table 1). The levels of HbA1c after 4 months decreased to 6.8+/−0.8%, 6.6+/−0.7% and 6.7+/−0.6%, in the oral, glargine and NPH insulin group respectively, without showing any significant differences between groups (p = 0.614; Table 2).

**Table 2:** **Patient characteristics after 4 months (visit 3)**

	**Oral**	**Glargine**	**NPH Insulin**	**all**	**p**
**N**	13	20	22	55	
**BMI (kg/m** ^**2**^ **)**	29.5+/−4.9	32.8+/−5.9	32.7+/−7.5	32.0+/−6.4	0.294
**HbA1c (%)**	6.8+/−0.8	6.6+/−0.7	6.7+/−0.6	6.7+/−0.7	0.614
**Fasting Glucose (mg/dl)**	139.5+/−36.0	136.5+/−49.9	119.4+/−32.4	130.2+/−40.5	0.264
**Serum Creatinin (mg/dl)**	0.8+/−0.2	0.9+/−0.2	0.9+/−0.2	0.9+/−0.2	0.647
**Triglycerides (mg/dl)**	166.5+/−93.8	178.7+/−112.8	205.2+/−203.2	186.4+/−150.9	0.741
**HDL-cholesterol (mg/dl)**	52.6+/−17.4	46.1+/−10.1	45.9+/−10.3	47.5+/−12.3	0.240
**LDL-cholesterol (mg/dl)**	106.2+/−31.6	85.7+/−38.4	105.3+/−25.2	98.3+/−33.0	0.108
**Hemoglobin (g/dl)**	14.0+/−1.0	13.7+/−1.4	14.2+/−1.5	14.0+/−1.3	0.494
**Leukocyte count /nl**	6.6+/−1.6	7.8+/−2.3	7.6+/−1.8	7.4+/−2.0	0.250
**Intima media thickness (mm)**	0.82+/−0.11	0.74+/−0.10	0.76+/−0.14	0.76+/−0.12	0.153
**Skin microvascular function (%)**	564.9+/−306.5	514.5+/−329.7	487.7+/−229.6	515.7+/−283.8	0.746

No significant differences between the groups concerning other metabolic parameters such as triglycerides, LDL and HDL cholesterol, body mass index or blood pressure could be observed. The number of reported adverse events including hypoglycemia, and serious adverse events did not differ between the groups.

A mean relative change of number of circulating EPC 4 weeks after start of therapy compared to baseline (as detected by FACS) of 1.6 (+/−4.2) was obtained in the intention to treat analysis set with median time between visit 1 and 2 of 28 days. The mean relative change was 1.4 (+/−4.1) in the glargine group, 1.5 (+/−4.7) in the NPH insulin group and 1.8 (+/−3.8) in the oral group. No statistically significant difference between the three groups could be shown. The global F-test yielded a p-value of 0.96. For that reason no post-hoc pairwise comparisons were performed. Analysis of the per protocol set yielded similar results (p = 0.95) (Table 3).

**Table 3:** **Relative change of EPC between visit 1–2 and visit 1–3 as detected in FACS analysis**

	**Oral**	**Glargine**	**NPH Insulin**	**all**	**p**
***EPC change visit 1-2***					
**N**	13	20	22	55	0.963
**Mean +/− SD**	1.8 +/−3.8	1.4 +/−4.1	1.5 +/−4.7	1.6 +/−4.2	
**Median (Q1, Q3)**	0.4 (−0.1, 1.0)	−0.2 (−0.7, 0.9)	−0.1 (−0.5, 0.8)	0.1 (−0.5, 0.9)	
***EPC change visit 1-3***					
**N**	13	19	21	53	0.551
**Mean +/− SD**	3.8 +/−9.5	1.3 +/−3.0	3.1 +/−7.4	1.3 +/−3.0	
**Median (Q1, Q3)**	1.0 (−0.4, 1.8)	0.2 (−0.7, 2.6)	0.2 (−0.2,1.7	0.2 (−0.4, 1.8)	

After four months of treatment, a mean relative change of 2.6 (+/−6.8) was obtained in the intention to treat analysis set with median time between visit 1 and 3 of 119 days. The mean relative change was 1.3 (+/−3.0) in the glargine group, 3.1 (+/−7.4) in the NPH insulin group and 3.8 (+/−9.5) in the oral group. No statistically significant differences between the three groups could be shown (p = 0.55, Table 3).

**Figure 2:**
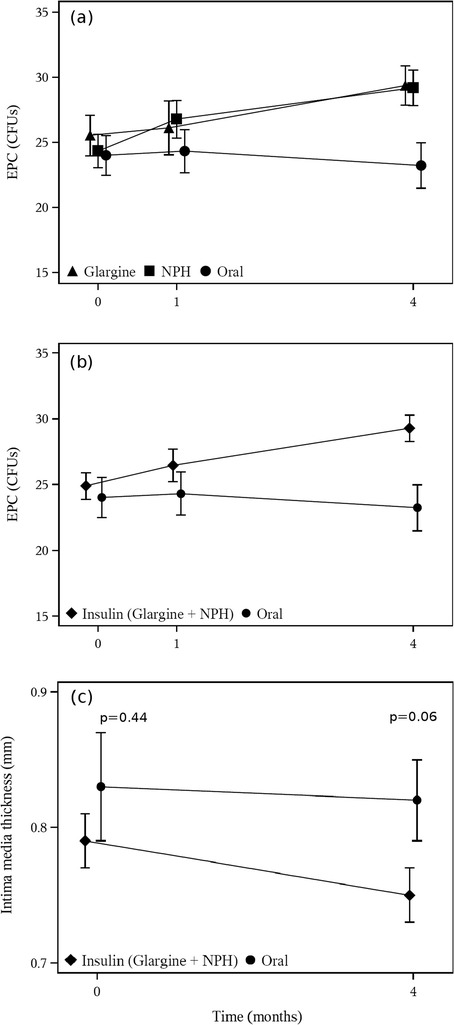
**Comparison of the number of colony forming units (CFUs) and intima media thickness between groups. (a)** Analysis shows significantly higher numbers in the NPH insulin group (29.2+/−6.4) and the glargine group (29.4+/− 6.7) compared to the oral group (23.2+/−6.3) after 4 months of treatment (p = 0.013). **(b)** Comparison of number of CFUs from all patients receiving insulin (NPH or glargine) to those on oral medication showing a significant effect after 4 months of treatment (p = 0.003). **(c)** Comparison of IMT from all patients receiving insulin (NPH or glargine) to those on oral medication. A trend towards a reduction of IMT after 4 months of insulin treatment could be documented (p = 0.06)

The number of colony forming units (CFUs) of EPC as detected by in vitro outgrowth were significantly higher in the NPH insulin group (29.2+/−6.4) and the glargine group (29.4+/−6.7) compared to the oral group (23.2+/−6.3) after 4 months (p = 0.013, Figure 2a). When data from all patients receiving insulin (NPH or glargine) were compared to those on oral medication a significant effect could be shown (p = 0.003, Figure 2b).

Mean intima media thickness (IMT) at visit 1 was 0.80 mm (+/−0.14) with mean IMT of 0.79 mm (+/−0.13), 0.79 mm (+/−0.17) and 0.83 mm (+/−0.13) in the glargine, NPH insulin and control group, respectively (p = 0.737, Table 1). At visit 3 mean IMT decreased to 0.76 mm (+/−0.12) with mean IMT of 0.74 mm (+/−0.10), 0.76 mm (+/−0.14) and 0.82 mm (+/−0.11) in the glargine, NPH insulin and control group, respectively (p = 0.153, Table 2) and therefore no statistical significance was shown. However, when after 4 months IMT data from all patients receiving insulin (NPH or glargine) were compared to those on oral medication a trend towards a reduction of IMT by insulin treatment could be documented (p = 0.06) indicating that in a longer trial or a larger study population a significant result might have been achieved (Figure 2c). Furthermore the decrease of IMT from 0.80 mm (+/−0.14) at visit 1 to 0.76 mm (+/−0.12) at visit 3 in all patients was statistically significant (p = 0.03) indicating that all patients profited from the intervention with respect to IMT.

Concerning the microvascular function of the skin at visit 1 a mean of 462.0% (+/−205.5) increase upon heat provocation could be observed, with a mean increase of 408.4% (+/−216.5), 455.7% (+/−171.8) and 555.1% (+/−223.5) in the glargine, NPH insulin and control group, respectively (Table 1). At visit 3 a mean of 515.7% (+/−283.8) increase upon heat provocation could be seen with a mean increase of 514.5% (+/−329.7), 487.7% (+/−229.6) and 564.9% (+/−306.5) in the glargine, NPH insulin and control group, respectively (Table 2). No significant difference between the three groups could be observed.

18 adverse events were reported. 5 in the glargine group, 7 in the NPH insulin group and 6 in the control group. Of those there were 2 mild hypoglyceamias reported in the glargine group. No adverse effects was categorized as severe.

## Discussion

This is the first study investigating the effects of add-on basal insulin treatment in a direct comparison to oral diabetes medication on endothelial progenitor cells in patients with type 2 diabetes mellitus. Our current data showed that a 4-month treatment with insulin glargine or NPH insulin significantly increases the ex vivo differentiation of EPCs in patients with type 2 diabetes mellitus, compared to an escalation of the oral antidiabetic therapy without additional insulin treatment. These findings are in agreement with our previously published data showing that insulin glargine and human insulin stimulate the clonogenic potential of EPCs in vitro [18]. However, no significant change in the numbers of circulating EPCs could be observed within the 4 months suggesting that there are no direct biological effects of insulin on the number of circulating EPC at least within the documented 4 months treatment in this study. These results are in agreement with a study by Fadini et al. where significant changes after adding insulin analogues in a crossover design could only be shown after 6 months of treatment [28]. As we previously described in a pilot study [17], a very recent study has shown an increase on circulating EPCs and outgrowth of CFUs in patients with long-standing uncontrolled type 2 diabetes receiving an intensified antidiabetic treatment. Yet this study did not compare different treatment strategies [29], which was the aim of our current study. Our data indicate that insulin treatment has a possible early effect on the functionality of EPCs while changes in numbers of circulating EPC could require more time and a long term improvement of metabolic control.

It has been previously shown that hyperglycemia reduces survival and impairs the function of EPCs in vitro [30] and that in patients with type 2 diabetes mellitus the levels of circulating EPCs are closely related to glycaemic control [31]. Poor glyceamic control is known to play a key role in the properties of circulating EPCs and several mechanisms seem to be involved in that, including advanced glycation end products formation [32] and reduced activity of silent information regulator 1 (SIRT1) and increased synthesis of platelet-activating factor (PAF) [33]. All 3 groups in this study achieved a significant decrease of HbA1c. However as aimed for, there were no significant differences concerning the HbA1c levels between the groups at baseline and after 4 months suggesting that the observed effects on EPC outgrowth did not depend on glycemic control but rather on direct insulin effects as we were previously able to show in vitro [18].

Since glargine and NPH insulin have a different structure and biologic activity, we assumed that the different pharmacokinetic properties could translate into different effects on EPCs. We were previously able to show that insulin stimulates the clonogenic potential of EPCs by IGF-1 receptor-dependent signaling [18] and it is known that insulin glargine has a 6-fold increased affinity for the IGF-1 receptor [34]. Yet the glargine metabolite gly-A21-insulin, that has been shown to have similar affinity to the IGF-1 receptors as human insulin [34], comprises most of the available substance in vivo [35]. Accordingly and in line with our previous in vitro data [18] we found no differences between NPH insulin and glargine on the function or the number of circulating EPCs. Furthermore no differences concerning other metabolic parameters as cholesterol levels, body mass index, skin microvascular function and in terms of safety profile including hypoglycemia could be observed.

IMT has been widely used as a surrogate parameter in primary intervention studies of cardiovascular risk reduction [36]. Previous studies have shown that patients with type 1 diabetes mellitus without macrovascular disease have low numbers of circulating EPCs and an increased IMT [37]. Moreover a previous study in middle-aged healthy people found a significant correlation between EPC number and IMT [38]. We were not able to detect any significant changes in carotid intima media thickness throughout the study between the 3 study groups. However, interestingly when IMT data from all patients receiving insulin (NPH or glargine) were compared to those on oral medication 4 months after baseline, a trend towards a reduction of IMT by insulin treatment could be documented indicating that in a trial with a larger study population a significant result might have been achieved. This hypothesis can be further supported by data recently published showing that in people with cardiovascular disease and/or CV risk factors, insulin glargine used to target normoglycemia modestly reduced carotid IMT progression [39].

Remarkably, at least a trend for a reduction of IMT was seen in patients treated with insulin while there were no effects of NPH insulin or glargine treatment on microvascular function. These data in our randomized and controlled setting contrasts previously published results from type 2 diabetes patients subjected to glycemic improvement by insulin or oral treatment [40–42]. It will have to be clarified, whether this is due to differences in study design or a consequence of methodological aspects. Yet, we believe that our data suggests that there could be only marginal effects of insulin treatment or improvement of glucose control since we did not see effects after 4 months of treatment even when patients in the study were pooled (data not shown).

Limitations of the study certainly include the relatively high rate of drop-outs which led to a reduction of the power of the study as mentioned above. Drop-out rates were relatively high mainly because patients that were randomized to receive insulin treatment would then decide to leave the study probably because they initially believed they would enter the oral medication arm although they were thoroughly instructed that this was a randomized trial. Additionally it should be noted that our current clinical study was based on a methodological paper that has been previously published [18]. Since then, different approaches have been used to culture and quantify EPC therefore the term EPCs should be used with caution since most commonly used methods for isolating EPCs in culture have been found to generate a mixed population of cells [43] However, we were previously able to show that the cells in our outgrowth assay carry established progenitor cell antigens, have a distinct influence on tube formation in vitro and incorporate into vascular structures and therefore should be considered angiogenic progenitors [18]. Even though the goal of this study was to detect possible differences on EPCs between patients on guideline-suggested diabetes oral medication or add-on therapy with insulin glargine and NPH insulin, the fact that in most cases the diabetes medication in the oral group was escalated could be a limitation since many oral antidiabetics have been known to have at least some effects on EPCs [44]. It is in most cases though not clear, whether these effects are direct drug effects or if they are a result of improved glycemic control. Furthermore several anti-diabetic and cardioprotective agents including metformin and rosuvastatin have been shown to have beneficial effects on endothelial cell viability, regeneration and apoptosis [45]. Even newer anti diabetic medication as the dipeptidyl peptidase-4 inhibitor Saxagliptin has been shown to improve the function of circulating pro-angiogenic cells from type 2 diabetic patients [46]. Glitazone treatment and a change in lipid lowering therapy, that were known at the time the study was designed to influence EPC, were not allowed in this study.

The results of this study are independent of glycemic control but nevertheless the functional meaning of an improved EPC differentiation still needs to be clarified.

## Conclusion

The study shows that a 4-month treatment with insulin glargine or NPH insulin significantly increased the outgrowth of EPCs in patients with type 2 diabetes mellitus compared to patients who received an escalation of the oral diabetes treatment only. The observed effects on EPC outgrowth did not depend on glycemic control but rather on direct insulin effects. We therefore believe that this study provides valuable information regarding pleiotropic effects of insulin that could possibly influence vascular homeostasis and outcomes.

## Abbreviations

CFUs: Colony-forming units
DPP4: Dipeptidyl peptidase-4
EPCs: Endothelial progenitor cells
FACS: Fluorescence-activated cell sorting
GOT: Glutamic oxaloacetic transaminase
GPT: Glutamic pyruvic transaminase
IGF-1: Insulin-like growth factor 1
IMT: Intima media thickness
ITT: Intention to treat
NPH: Neutral protamin Hagedorn
ORIGIN: Outcome reduction with initial glargine intervention
pBMCs: Peripheral blood mononuclear cells
PP: Per protocol
PU: Perfusion units
UKPDS: United Kingdom prospective diabetes study
VEGF-2: Vascular Endothelial Growth Factor 2
